# LC-MS/MS Analysis and Pharmacokinetics of Sodium (±)-5-Bromo-2-(α-hydroxypentyl) Benzoate (BZP), an Innovative Potent Anti*-*Ischemic Stroke Agent in Rats

**DOI:** 10.3390/molecules21040501

**Published:** 2016-04-16

**Authors:** Xin Tian, Bingjie Liu, Yuhai Zhang, Hongmeng Li, Jingyao Wei, Gaoju Wang, Junbiao Chang, Hailing Qiao

**Affiliations:** 1 Institute of Clinical Pharmacology, Zhengzhou University, Zhengzhou 450052, Henan, China; tianx@zzu.edu.cn (X.T.); jimlee_2007@126.com (H.L.); 15981980136@163.com (J.W.); wanggaoju@gs.zzu.edu.cn (G.W.); 2 Department of Pharmacy, The First Affiliated Hospital of Zhengzhou University, Zhengzhou 450052, Henan, China; 3 College of Chemistry and Molecular Engineering, Zhengzhou University, Zhengzhou 450001, Henan, China; bingjiewait@163.com (B.L.); zhangyh228@163.com (Y.Z.)

**Keywords:** BZP, metabolite, Br-NBP, LC-MS/MS, pharmacokinetics, rat plasma

## Abstract

A rapid, sensitive and selective liquid chromatography-triple quadrupole mass spectrometry (LC-MS/MS) method was developed and validated for the simultaneous determination of sodium (±)-5-bromo-2-(α-hydroxypentyl) benzoate (BZP) and its active metabolite 3-butyl-6-bromo-1(3*H*)-isobenzofuranone (Br-NBP) in rat plasma using potassium 2-(1-hydroxypentyl)-benzoate (PHPB) and l-3-n-butylphthalide (NBP) as internal standards (IS). Chromatographic separation was achieved on a Hypersil GOLD C18 column using a gradient elution of ammonium acetate and methanol at a flow rate of 0.2 mL/min. Good linearity was achieved within the wide concentration range of 5–10,000 ng/mL. The intra-day and inter-day precision was less than 8.71% and the accuracy was within −8.53% and 6.38% in quality control and the lower limit of quantitation samples. BZP and Br-NBP were stable during the analysis and the storage period. The method was successfully applied to pharmacokinetic studies of BZP in Sprague-Dawley rats for the first time. After a single intravenous administration of BZP at the dose of 0.75 mg/kg, the plasma concentration of BZP and Br-NBP declined rapidly and the *AUC*_0-t_ of BZP was significantly greater in female rats compared to male rats (*p* < 0.05). The data presented in this study serve as a firm basis for further investigation of BZP in both preclinical and clinical phases.

## 1. Introduction

**Figure 1:**
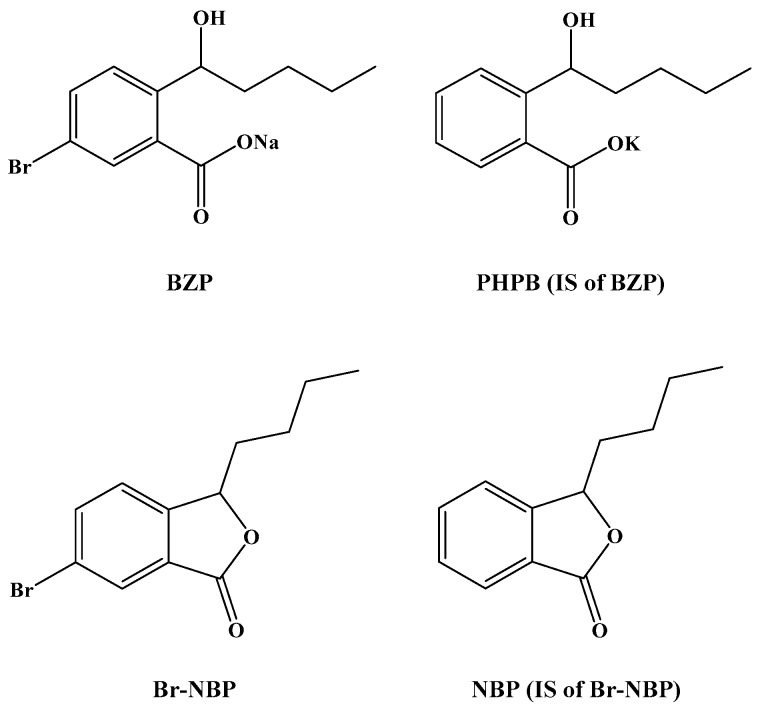
Chemical structures of BZP, PHPB (IS of BZP), Br-NBP and NBP (IS of Br-NBP).

Ischemic stroke accounts for 75%–85% of all the strokes occurring annually in China, which is a leading cause of death and functional limitations worldwide [1,2,3]. Extracted from the seeds of Apium graveolens Linn, l-3-n-butylphthalide (NBP) was developed as a neuroprotective agent against cerebral ischemia [4]. Brozopentyl sodium salt, sodium (±)-5-bromo-2-(α-hydroxypentyl) benzoate (BZP) (Figure 1), derived from NBP, is a novel agent that shows promising outcomes for the treatment of ischemic stroke.

NBP treatment significantly lowered neurological deficit scores, reduced infarct volume, and minimized pathological changes in the penumbra area in middle cerebral artery occlusion (MCAO) rats [5]. The mechanisms underlying neuroprotective effects of NBP may be due to activating protein kinase A (PKA) and protein kinase B (AKt) prosurvival pathways and inhibiting microglia activation [6,7]. In addition, NBP can play an efficient role in attenuating amyloid-induced cell death in neuronal cultures, improving cognitive impairment in an animal model of Alzheimer’s disease and preventing neuronal cell death after focal cerebral ischemia in mice via the c-Jun N-terminal kinase pathway in recent studies [8,9,10]. However, the toxic reactions may arise due to its metabolites’ higher plasma exposures than that of NBP [11]. The bioavailability being as low as 15% limits the application of NBP capsules in acute ischemic stroke patients [12].

A series of NBP derivatives were designed and synthesized in order to improve their pharmacological activity. The pharmacological activity of all compounds have been evaluated *in vitro* and *in vivo*. Our prior study [13,14] showed that 3-butyl-6-bromo-1(3*H*)-isobenzofuranone (Br-NBP) had anti-hydrogen peroxide-induced damage in PC12 cells and anti-platelet aggregation effect. However, it is difficult to prepare an intravenous injection formulation as the water-solubility of Br-NBP is poor. Therefore, BZP has been synthesized in order to improve Br-NBP’s water-solubility. In addition, BZP can be converted to Br-NBP *in vivo* and *in vitro*. Our previous studies showed that BZP and Br-NBP had the ability to reduce cerebral infarction volume in the focal cerebral ischemia-reperfusion injury and prevent permanent global brain ischemia, but BZP proved to be more active and secure than Br-NBP. Its mechanism could be partly explained by stating that BZP can inhibit the mitochondrial apoptotic pathways and enhance the neuro-plasticity [15].

Therefore, BZP was approved for clinical trials by the State Food and Drug Administration (SFDA) of China. In order to explore pharmacokinetic and pharmacological characters of BZP and Br-NBP, it is essential to develop an analytical method for simultaneous determination of BZP and Br-NBP in biological samples. In the present work, a sensitive and reproducible liquid chromatography coupled to the mass spectrometry (LC-MS/MS) method for determination of BZP and Br-NBP in rat plasma was developed and validated. Then, based on the developed method, we evaluated the pharmacokinetics of BZP and Br-NBP in rats after a single dose intravenous administration.

## 2. Results and Discussion

### 2.1. Optimization of the Sample Treatment

Different approaches were investigated as a simple protein precipitation by three organic solvents (methanol, ethanol and acetonitrile). The results showed that the recovery was acceptable when precipitating plasma with the three organic solvents. However, the matrix effect while using methanol as the precipitant was better than ethanol and acetonitrile. At a volume ratio of 4:1, methanol showed a high extraction recovery and resulted in much cleaner extracts. Therefore, methanol was used as the protein-precipitating agent at a volume ratio of 4:1 in further experiments.

### 2.2. Optimization of the Mass Parameters

The MS/MS operation parameters were carefully optimized for the determination of the analytes. As the polar of Br-NBP was low, Br-NBP and NBP exhibited a stronger mass response in positive ionization mode. Br-NBP and NBP showed predominant protonated precursor [M + H]^+^ ions at *m*/*z* 269 and 191, respectively. However, BZP and Potassium 2-(1-hydroxypentyl)-benzoate (PHPB) display higher signal intensities in (−) ESI mode than in (+) ESI mode as the polar of BZP was high. BZP and PHPB displayed an [M − H]^−^ ion at *m*/*z* 285 and 207. The selected reaction monitoring (SRM) mode was used for its better specificity and sensitivity relative to the selected ion monitoring (SIM) mode. The product ion scan spectra showed high abundance fragment ions at *m*/*z* 85, 85, 144 and 145 for BZP, PHPB, Br-NBP and NBP, respectively. Therefore, multiple reaction monitoring (MRM) reaction of BZP, PHPB, Br-NBP and NBP at *m*/*z* 285→85, *m*/*z* 207→85, *m*/*z* 269→144, *m*/*z* 191→145 were used for quantification. The MS/MS tuning was tested by selecting a negative ionization mode before 5 min and by selecting a positive ionization mode at 5–8 min. Parameters such as the spray voltage, capillary temperature, sheath gas pressure, auxiliary gas pressure, vaporizer temperature and collision energy were automatically optimized using the TSQ Tune Master program (Thermo Fisher Scientific Inc., Waltham, MA, USA). A summary of the optimized SRM fragmentation transitions and MS parameters for each analyte is reported in Table 1.

### 2.3. Selection of Chromatographic Parameters and the Internal Standard

A series of screenings for HPLC columns were investigated, including a Zorbax SB-CN column (150 mm × 4.6 mm, 3 μm) (Agilent Technologies, Delaware, CA, USA), a YMC-Pack Pro C18 column (150 mm × 4.6 mm, 3 μm) (YMC Karasuma-Gojo Bldg., Kyoto, Japan) and a Hypersil GOLD C18 column (100 mm × 2.1 mm, 5 μm) (Thermo Fisher Scientific Inc., Waltham, MA, USA). Hypersil GOLD C18 (100 mm × 2.1 mm, 5 μm) exhibited the best separation. Furthermore, several different percentages of methanol/water and acetonitrile/water, as well as the pH of the buffer solution, were investigated for the mobile phase. Based on its stability, BZP was better in neutral than in acidic and alkaline environments. Finally, a gradient mobile phase consisting of 5 mM ammonium acetate and methanol was used to obtain symmetrical and sharp peaks. PHPB and NBP were used as internal standard (IS) and were suitable for quantitative analysis with a high recovery and a low matrix effect. BZP and PHPB were eluted at 3.34 and 2.14 min, Br-NBP and NBP were eluted at 6.56 and 5.56 min, respectively, with total run time of 8 min.

### 2.4. Method Validation

#### 2.4.1. Specificity

**Figure 2:**
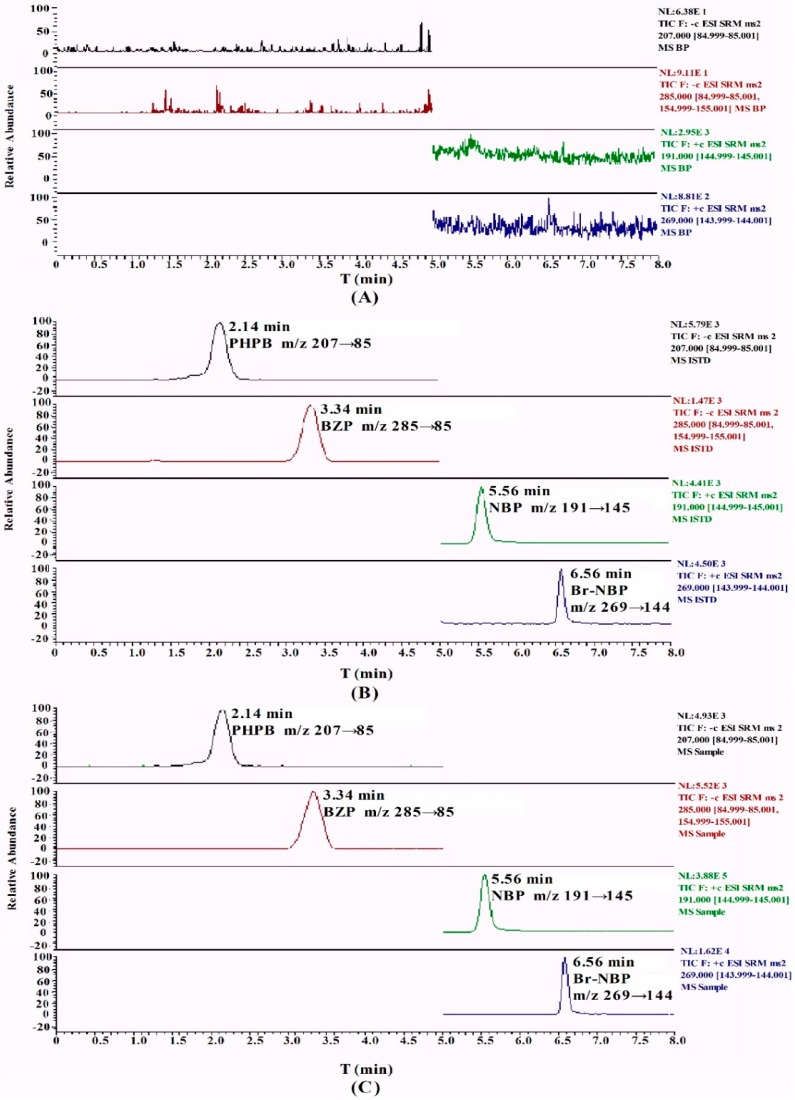
Representative chromatograms of BZP, PHPB (IS of BZP), Br-NBP and NBP (IS of Br-NBP) in (**A**) blank plasma; (**B**) plasma spiked with BZP, Br-NBP and IS; and (**C**) sample after an intravenous administration of BZP. The retention times for BZP, PHPB, Br-NBP and NBP were 3.34, 2.14, 6.56 and 5.56 min, respectively.

Figure 2 shows the typical chromatograms of blank plasma, plasma spiked with BZP and Br-NBP, and the sample collected from rats after intravenous administration of BZP. No significant interfering peaks were observed at the retention time of BZP, Br-NBP and IS. The peaks of BZP, Br-NBP and IS were detected with high resolution and excellent peak shape.

#### 2.4.2. Assay Linearity and LLOQ

The calibration curves showed excellent linearity over ranges examined, with correlation coefficients (r^2^) greater than 0.99. The weighing factor 1/χ^2^ was selected for back calculation of the nominal value because it produced the best linear fit. In addition, the signal intensity of the lower limit of quantification (LLOQ) sample was five times stronger compared with the blank plasma signal. Calibration standards (CSs) were made by spiking blank plasma with appropriate volumes of working solutions. Final calibration standard concentrations for BZP/Br-NBP were 5, 10, 50, 250, 1000, 5000, and 10,000 ng/mL. The linear equations of BZP and Br-NBP were Y = −0.0016 + 0.0056X (r^2^ = 0.9937) and Y = 0.0008 + 0.0001X (r^2^ = 0.9911), respectively, where Y is peak area ratio (BZP/IS, Br-NBP/IS), and X represents concentrations of BZP and Br-NBP (ng/mL). The LLOQ of both BZP and Br-NBP in plasma were determined to be 5 ng/mL with acceptable accuracy and precision.

#### 2.4.3. Precision and Accuracy

The intra-day and inter-day precision and accuracy data of BZP and Br-NBP for quality control samples (QCs) and LLOQ in plasma are presented in Table 2. The intra-day and inter-day precision (RSD, %) values of BZP were less than 7.12% and 6.79%, respectively, and the intra-day and inter-day accuracy (%) were in the range of −5.47%–2.83% and −8.39%–5.57%, respectively. For Br-NBP, the intra-day and inter-day precision ranged from 0.70%–5.97% and 0.64%–8.71%, respectively, while the intra-day and inter-day accuracy were in the ranges of −1.77%–3.70% and −8.53%–6.38%, respectively. All these results were within the criteria set by regulatory guidelines, which indicated that the present method has good accuracy, precision and reproducibility.

#### 2.4.4. Recovery and Matrix Effects

The extraction recovery and matrix effects for BZP and Br-NBP (at three different QC levels) in plasma were shown in Table 3. The mean recoveries of BZP and PHPB in the rat plasma were greater than 106.3% and 89.5%, respectively. Furthermore, the mean recoveries of Br-NBP and NBP were greater than 99.5% and 88.2%, respectively. The mean matrix effects of BZP and PHPB in the rat plasma were from 103.0% to 110.1% and 101.8%, respectively. The mean matrix effects of Br-NBP and NBP were from 99.7% to 109.1% and 106.3%, respectively. The results proved that recovery of each compound was consistent, precise and reproducible and there was insignificant matrix effect.

#### 2.4.5. Stability

Stability was investigated under different conditions. As illustrated in Table 4, BZP and Br-NBP were found to be stable in the rat plasma under various storage conditions with acceptable accuracy and precision, which demonstrated that BZP and Br-NBP have a good stability for pharmacokinetic studies.

#### 2.4.6. Sample Dilution

To demonstrate the capacity of diluting plasma samples containing BZP and Br-NBP at concentrations above the upper limit of quantification (ULOQ), a set of plasma samples were prepared containing BZP and Br-NBP at concentrations of 10,000, 50,000 and 150,000 ng/mL and stored at −80 °C overnight prior to analysis. After thawing at room temperature, the spiked samples were diluted with blank rat plasma to generate the final concentrations of 500, 2500 and 7500 ng/mL. The results of sample dilution are shown in Table 5, which demonstrated the diluting high concentration samples with blank plasma could not affect the accuracy and precision of the assay.

### 2.5. Application to Pharmacokinetic Study

**Figure 3:**
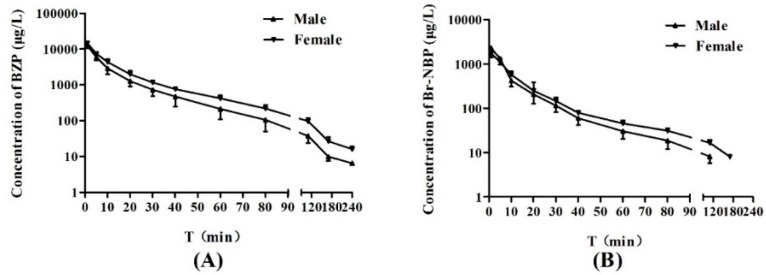
Mean plasma concentration-time profiles of BZP and Br-NBP (each point represents mean ± SD). (**A**) mean plasma concentration-time profiles of BZP after intravenous administration of BZP at a single dose of 0.75 mg/kg in SD rats; (*n* = 10); (**B**) mean plasma concentration-time profiles of Br-NBP after intravenous administration of BZP at a single dose of 0.75 mg/kg in SD rats; (*n* = 10).

After administration of BZP (0.75 mg/kg) to rats, the mean plasma concentration-time profiles and summary of the pharmacokinetic parameters of BZP and Br-NBP are presented in Figure 3 and Table 6. The result indicated that the area under the plasma concentration–time curve (*AUC*_0-t_), the peak plasma concentration (*C*_1min_) and biological half-life (*t*_1/2_) for BZP were 148.21 ± 34.16 (mg·h/L), 13,527 ± 2120 (ng/mL) and 27.21 ± 2.41 (min), respectively. The *AUC*_0-t_ of BZP was significantly greater in female rats compared to male rats (*p* < 0.05). However, there was no gender difference in rats for the other PK parameters of BZP.

Formation of Br-NBP was rapid with maximum plasma concentration of 2024.02 ± 650.17 (ng/mL) at 1 min after intravenous administration of BZP (0.75 mg/kg). The results indicated that the biotransformation of BZP to Br-NBP in rat plasma was rapid and extensive. Furthermore, the average ratio of *AUC*_0-t_ (BZP) to *AUC*_0-t_ (Br-NBP) were 4.94 for male rats and 6.87 for female rats, which indicated that transformation ability was different in male and female rats (*p* < 0.05). The *AUC*_0-t_ and *t*_1/2_ for Br-NBP were 21.68 ± 2.54 (mg·h/L) and 24.92 ± 5.23 (min), respectively. There was no gender difference in rats for all the PK parameters of Br-NBP.

## 3. Experimental Section

### 3.1. Chemical and Reagents

BZP bulk drug (purity 99.4%) and Br-NBP (purity 99.8%) were discovered and synthesized at the College of Chemistry and Molecular Engineering, Zhengzhou University (Zhengzhou, China). PHPB (internal standard, IS, purity 98.5%) was synthesized at College of Chemistry and Molecular Engineering, Zhengzhou University. NBP (internal standard, IS, purity 99.5%) was purchased from CSPC NBP Pharmaceutical Co., Ltd. (Shijiazhuang, China). Methanol (HPLC grade) was obtained from Fisher, (Waltham, MA, USA). Ammonium acetate (analytically pure) was purchased from Sinopharm Chemical Reagent Co., Ltd. (Shanghai, China). Purified water from a Milli-Q system (Millipore, Bedford, MA, USA) was used throughout. All other chemicals were of analytical grade and used without further purification.

### 3.2. Calibration Standard and Quality Control Samples

BZP, Br-NBP and mixed IS (consisting of PHPB and NBP) working standards were weighed accurately and transferred into a 10 mL volumetric flask. Analytes were dissolved in 2 mL of methanol, and the volume was made for up to 10 mL with methanol. Final concentration of BZP, PHPB (IS of BZP), Br-NBP and NBP (IS of Br-NBP) were all at 100 µg/mL, respectively. Analytical working standards were prepared with methanol by serial dilutions from the stock solution.

Calibration standards (CSs) and QC samples were made by spiking blank plasma with appropriate volumes of working solutions. Final calibration standard concentrations for BZP/Br-NBP were 5, 10, 50, 250, 1000, 5000, 10,000 ng/mL. The QC solutions were prepared by spiking QC working solutions into blank plasma to produce QCs of 5000 (high), 250 (medium) and 10 (low) ng/mL. The mixed IS (consisting of PHPB and NBP) solution (250 ng/mL) was prepared by diluting the stock solution in methanol. All stock solutions and working solutions were stored at 4 °C and brought to room temperature before use.

### 3.3. Instrumentation and Analytical Conditions

The quantification of BZP and Br-NBP was performed on the LC-MS/MS system consisting of a Thermo Fisher ACCELA LC system connected with a Thermo Fisher TSQ QUANTUM ULTRA triple-quadrupole mass spectrometer with an ESI interface (Thermo Fisher Scientific Inc., Waltham, MA, USA). The chromatographic separation was achieved on Hypersil GOLD C18 (100 mm × 2.1 mm, 5 μm) maintained at 30 °C temperature at a flow rate of 0.2 mL/min. Elution was performed by a gradient mobile phase consisting of 5 mM ammonium acetate (A) and methanol (B). The gradient started at 55% B for 2 min, increasing linearly to 90% B within 2 min, then maintained at 90% B for another 1 min, after which the level of B was decreased back to 55% within 0.1 min, then the column was equilibrated till 8 min prior to next sample injection. The injection volume was 10 μL in no waste mode.

The mass spectrometer was operated in (−) ESI mode for the first 5 min and switched to (+) ESI mode in 5–8 min. Quantitation was performed by the SRM mode. The parameter settings were as follows: spray voltage of positive-ion, 3500 V; spray voltage of negative-ion, 2500 V; capillary temperature, 350 °C; sheath gas (nitrogen), 30 Arbitrary units; auxiliary gas (nitrogen), eight Arbitrary units; vaporizer temperature, and room temperature. Thermo Fisher LCQUAN quantitative software (Thermo Fisher Scientific Inc.) was used.

### 3.4. Sample Preparation

A simple protein precipitation method was applied to extract the analyte from the plasma sample. An aliquot of 50 μL of plasma was pipetted into a 1.5 mL capped polypropylene tube, and then 50 μL IS working solution and 150 μL methanol were spiked and mixed by vortexing for 60 s. After centrifuging at 13,000× rpm for 10 min, 200 μL supernatant was transferred into a 1.5 mL capped polypropylene tube and centrifuged at 13,000× rpm for 5 min again. After centrifugation, the supernatant was transferred to a fresh vial and 10 μL was injected into a chromatographic system for analysis.

### 3.5. Method Validation

The method was validated for selectivity, linearity, precision, accuracy, extraction recovery, matrix effects and stability according to the USA Food and Drug Administration (FDA) guidelines for the validation of bioanalytical methods [16].

#### 3.5.1. Specificity

The specificity of the method was investigated by analyzing blank plasma samples from six different rats, blank plasma spiked with standard solution and plasma samples collected from rats after intravenous administration of BZP.

#### 3.5.2. Lower Limit of Quantitation and Linearity

LLOQ was established using six samples independent of the standards and was determined with precision less than 20% and accuracy within ±20%. Plasma calibration standards of BZP and Br-NBP were prepared and assayed on three separate days. Calibration curves were constructed using the peak area ratios of each analyte to IS *versus* concentrations of analyte in plasma. The linearity was assessed by a weighted (1/χ^2^) least squares linear regression in the range of 5 to 10,000 ng/mL. The acceptance criterion was that the coefficient of correlation (R) was greater than 0.99, and the residuals were within ±15% at all other calibration levels except at LLOQ, which was set at ±20%.

#### 3.5.3. Precision and Accuracy

The intra-day precision and accuracy were evaluated by analyzing six replicate QC samples at concentrations of 10, 250 and 5000 ng/mL on the same day. To determine the inter-day accuracy and precision, analysis of three batches of QC samples were performed on different days. The acceptance criteria included accuracy within 15% of the nominal concentration and a precision that does not exceed 15% of the relative standard deviation (RSD).

#### 3.5.4. Extraction Recovery and Matrix Effects

Plasma extraction recovery and matrix effect for each tested compound were evaluated by analyzing samples at concentrations of 10, 250 and 5000 ng/mL. The recovery was evaluated by comparing the peak area of extracted QC samples with corresponding peak areas for the blank plasma extracted and spiked with BZP, Br-NBP and IS after extraction at equivalent concentrations (*n* = 3).

To evaluate the matrix effect, blank rat plasma was extracted and then spiked with BZP, Br-NBP and IS at concentrations of 10, 250 and 5000 ng/mL. The corresponding peak areas were then compared to those of neat standard solutions at equivalent concentrations, and this peak area ratio is defined as the matrix effect (*n* = 3).

#### 3.5.5. Stability

Stability studies were assessed by analyzing three replicates of the QC samples at three concentrations. The short-term stability was evaluated after exposure of the samples to ambient temperature for 4 h. The long-term stability in plasma was assessed by analyzing the QC samples stored at −80 °C for 1 month. The auto-sample stability was assessed by keeping at auto-sample (25 °C) for 24 h. The freeze and thaw stability were evaluated by exposing QC samples to three freeze (−80 °C) and thaw cycles before sample preparation. In each cycle, the samples were thawed at room temperature for 30 min and refrozen for 24 h. The room temperature stability for stock solution was evaluated after storing the sample at 25 °C for 24 h. The 4 °C stability for stock solution was evaluated after storing the sample at 4 °C for 1 month.

#### 3.5.6. Dilution Methods

An aliquot of 45 μL of plasma was pipetted into a 1.5 mL capped polypropylene tube, then 5 μL BZP (1500, 500, 100 μg/mL) and Br-NBP (1500, 500, 100 μg/mL) working solution were spiked and mixed by vortexing for 60 s. Then, 5 μL of medicated plasma was removed out and mixed with 95 μL blank plasma by vortexing for 60 s. Samples of dilution plasma were dealt with according to “2.4 sample preparation” and the concentrations of BZP and Br-NBP were measured.

### 3.6. Animal Experimentation

Sprague–Dawley (SD) rats, weighing 200–240 g, half male and half female, were provided by Beijing Vital River Laboratory Animal Technology Co., Ltd. (Beijing, China). Animals were housed under controlled environmental conditions (ambient temperature 23–25 °C; 12 h light/12 h darkness cycle, 45%–55% relative humidity) and maintained on standard pellet diet and water *ad libitum* throughout the experimental period. The animals were fasted overnight with free access to water for at least 12 h before administration. This study was performed according to the Guide for the Care and Use of Laboratory Animals. All experimental procedures reported herein were reviewed and approved by the Zhengzhou University Animal Care and Use Committee.

### 3.7. Pharmacokinetic Studies

The 10 mg/mL solution of BZP was prepared by dispersing in 0.9% saline solution. Ten rats (*n* = 10, each group, half male and half female) received a single intravenous (i.v.) administration of BZP at 0.75 mg/kg via tail vein. Blood samples (200 μL) for pharmacokinetic analyses were collected pre-dose and at 1, 5, 10, 20, 30, 40, 60, 80, 110, 170 and 240 min post-BZP dose by orbital bleeding via heparinized capillary tubes.

Plasma samples were harvested by centrifuging the blood at 4500 rpm for 10 min at 4 °C and stored frozen at −80 °C until analysis.

### 3.8. Pharmacokinetic Calculation and Statistical Analysis

The concentration *versus* time data and the pharmacokinetic parameters including *C_max_*, *t_1/2_*, *AUC*_0-t_, *AUC*_0-∞_, *MRT*_0-t_, *MRT*_0-∞_, *CL_z_* and *V_z_* were assessed via non-compartmental analysis using the DAS 2.0 package (version 2.0 pharmacokinetic software; Chinese pharmaco-logical Assn., Beijing, China). An independent-samples *t*-test was analyzed twice to evaluate the differences of pharmacokinetic parameters between the two groups. The data were expressed as the mean ± standard deviation (SD). A value of *p* < 0.05 was considered to be statistically significant. All statistical analyses were performed with SPSS 17.0 for Windows (SPSS Inc., Chicago, IL, USA).

## 4. Conclusions

In this study, a new rapid, sensitive and reliable LC-MS/MS method was established and validated for simultaneous determination of BZP and its metabolite Br-NBP in rat plasma for the first time. This method proved to be superior with respect to its excellent selectivity, small sample volume, simple sample preparation and sensitivity with LLOQ of BZP and Br-NBP at 5 ng/mL. The linearity is good over the linear range of 5–10,000 ng/mL to investigate the BZP and Br-NBP in SD rat plasma. Unknown sample concentrations exceeding the range were diluted and re-assayed. The method was successfully applied to the pharmacokinetic study of BZP, which will be helpful in providing a firm basis and useful information for further research into the preclinical and clinical pharmacokinetics of BZP.

## Figures and Tables

**Table 1:** The MS parameters of BZP, PHPB, Br-NBP and NBP. (+, positive; −, negative).

Compound	ESI Mode	Retention Time (min)	SRM	Collision Energy (eV)
BZP	−	3.34	285→85	19
PHPB	−	2.14	207→85	19
Br-NBP	+	6.56	269→144	18
NBP	+	5.56	191→145	14

**Table 2:** Summary of precision and accuracy for BZP and Br-NBP in Sprague-Dawley (SD) rat plasma.

Compound	Spiked	Intra-Day (*n* = 6)			Inter-Day (*n* = 18)		
		Found Con. (Mean ± S.D.) (ng/mL)	Precisin (RSD) %	Accuracy (%)	Found Con. (Mean ± S.D.) (ng/mL)	Precisin (RSD) %	Accuracy (%)
BZP	5.0 (LLOQ)	5.14 ± 0.37	7.12	2.83	5.28 ± 0.16	3.13	5.57
	10.0 (QC-low)	9.45 ± 0.33	3.53	−5.47	9.16 ± 0.42	4.61	−8.39
	250.0 (QC-medium)	252.65 ± 2.94	1.16	1.06	252.97 ± 13.21	5.22	1.19
	5000.0 (QC-high)	5127.92 ± 283.49	5.53	2.56	4838.89 ± 328.34	6.79	−3.22
Br-NBP	5.0 (LLOQ)	5.03 ± 0.25	4.94	0.60	5.03 ± 0.03	0.64	0.61
	10.0 (QC-low)	10.18 ± 0.07	0.70	1.76	10.09 ± 0.68	6.76	0.95
	250.0 (QC-medium)	259.25 ± 5.81	2.24	3.70	265.96 ± 13.86	5.21	6.38
	5000.0 (QC-high)	4911.53 ± 293.07	5.97	−1.77	4573.30 ± 398.18	8.71	−8.53

**Table 3:** The recovery and matrix effect of BZP and Br-NBP in SD rat plasma (*n* = 3).

Compound	Spiked Concentration (ng/mL)	Recovery (Mean ± S.D.) %	RSD (%)	Matrix Effect (Mean ± S.D.) %	RSD (%)
BZP	10.0 (QC-low)	107.1 ± 1.1	1.0	103.0 ± 0.3	0.3
	250.0 (QC-medium)	108.3 ± 1.6	1.4	110.1 ± 1.0	0.9
	5000.0 (QC-high)	106.3 ± 1.5	1.4	109.0 ± 0.1	0.1
PHPB	250.0 (IS)	89.5 ± 0.5	0.5	101.8 ± 10.3	10.1
Br-NBP	10.0 (QC-low)	104.0 ± 0.7	0.6	99.7 ± 3.6	3.6
	250.0 (QC-medium)	99.5 ± 0.4	0.4	109.1 ± 1.7	1.6
	5000.0 (QC-high)	102.1 ± 1.3	1.2	105.4 ± 4.5	4.2
NBP	250.0 (IS)	88.2 ± 0.3	0.3	106.3 ± 4.7	4.4

**Table 4:** Stability of BZP and Br-NBP in SD rat plasma (*n* = 3).

Storage Conditions	Nominal Conc. (ng/mL)	BZP			Br-NBP		
		Determined Conc. (Mean ± S.D., ng/mL)	RE (%)	RSD (%)	Determined Conc. (Mean ± S.D., ng/mL)	RE (%)	RSD (%)
Short time stability ^a^	10.0 (QC-low)	10.53 ± 0.44	5.33	4.15	10.11 ± 0.65	1.12	6.42
	250.0 (QC-medium)	264.78 ± 14.10	5.91	5.33	266.65 ± 8.20	6.66	3.08
	5000.0 (QC-high)	5247.95 ± 339.51	4.96	6.47	5054.63 ± 391.31	1.09	7.74
Long time stability ^b^	10.0 (QC-low)	9.35 ± 0.84	−6.54	8.96	11.36 ± 0.48	13.62	4.32
	250.0 (QC-medium)	257.55 ± 0.81	3.02	0.32	277.53 ± 1.77	11.01	0.64
	5000.0 (QC-high)	5457.37 ± 10.10	9.15	0.19	5542.47 ± 81.30	10.85	1.47
Auto-sample stability ^c^	10.0 (QC-low)	10.29 ± 1.29	2.92	12.49	9.83 ± 0.56	−1.74	5.69
	250.0 (QC-medium)	257.41 ± 7.99	2.96	3.10	274.28 ± 7.33	9.74	2.67
	5000.0 (QC-high)	5422.81 ± 147.65	8.46	2.72	5563.03 ± 80.51	11.26	1.45
Freeze-thaw stability ^d^	10.0 (QC-low)	10.14 ± 0.64	1.41	6.27	9.51 ± 1.41	−4.94	14.80
	250.0 (QC-medium)	255.94 ± 7.52	2.38	2.94	269.64 ± 3.02	7.86	1.12
	5000.0 (QC-high)	5424.83 ± 295.27	8.50	5.44	5237.06 ± 363.48	4.74	6.94
Room temperature Stability for stock Solution ^e^	10.0 (QC-low)	9.61 ± 0.34	−3.94	3.54	8.81 ± 0.48	−11.91	5.43
	250.0 (QC-medium)	227.43 ± 7.43	−9.03	3.27	278.46 ± 4.52	11.38	1.62
	5000.0 (QC-high)	5204.52 ± 181.16	4.09	3.48	5741.23 ± 141.56	14.82	2.47
At 4 °C stability for Stock solution ^f^	10.0 (QC-low)	9.61 ± 1.09	−3.94	11.33	10.3 ± 0.62	3.31	5.98
	250.0 (QC-medium)	260.75 ± 9.26	4.30	3.55	272.94 ± 5.76	9.17	2.11
	5000.0 (QC-high)	5302.79 ± 31.30	6.06	0.59	5266.6 ± 201.45	5.33	3.82

^a^ Exposed at ambient temperature (25 °C) for 4 h; ^b^ Stored at −80 °C for 1 month; ^c^ Kept at auto-sample (25 °C) for 24 h; ^d^ After three freeze-thaw cycles; ^e^ Stored at 25 °C for 24 h; ^f^ Stored at 4 °C for 1 month.

**Table 5:** Summary of dilution effects of BZP and Br-NBP in SD rat plasma (*n* = 6).

Dilution Factor	Compound	Assayed Concentration (Mean ± S.D.) (ng/mL)	Reported Concentration (Mean ± S.D.) (ng/mL)	RSD (%)	Accuracy (%)
20	BZP	445.45 ± 7.90	8909.19 ± 158.35	4.25	5.22
		2338.05 ± 65.35	46761.27 ± 1306.70	1.78	−10.91
		7891.70 ± 335.35	157,833.80 ± 6706.90	2.79	−6.48
	Br-NBP	471.40 ± 7.45	9428.13 ± 148.50	2.48	−10.25
		2275.80 ± 82.25	45,515.87 ± 1644.95	1.58	−5.72
		6731.40 ± 167.15	134,628.10 ± 3342.71	3.61	−8.97

Nominal concentration: 10,000, 50,000 and 150,000 ng/mL for BZP and Br-NBP.

**Table 6:** Pharmacokinetic parameters of BZP and Br-NBP in SD rat plasma (*n* = 10).

Parameter	BZP		Br-NBP	
	Male	Female	Male	Female
*AUC*_0-t_ (mg·h/L)	122.46 ± 24.81 *	173.44 ± 19.67	21.48 ± 6.32	21.88 ± 2.54
*AUC*_0-∞_ (mg·h/L)	122.69 ± 24.78 *	173.72 ± 19.59	21.84 ± 6.28	22.45 ± 2.75
*MRT*_0-t_ (min)	14.52 ± 3.21	20.13 ± 1.96	11.75 ± 1.33	16.58 ± 2.79
*MRT*_0-t_ (min)	14.87 ± 3.08	20.50 ± 1.91	13.72 ± 1.24	19.85 ± 2.89
*t*_1/2z_ (min)	22.56 ± 6.32	27.21 ± 2.41	22.78 ± 3.34	27.06 ± 6.23
*CL_z_* (L/min·kg)	0.006 ± 0.002	0.004 ± 0.001	0.037 ± 0.010	0.034 ± 0.004
*V_z_* (L/kg)	0.21 ± 0.08	0.17 ± 0.02	1.235 ± 0.511	1.294 ± 0.200
*C*_1min_ (ng/mL)	12435 ± 1920	14620 ± 1855	2290.18 ± 868.91	1757.86 ± 137.91

Data are given as the mean ± standard deviation (SD); *AUC*: the area under the plasma concentration-time curve; *MRT*: mean residence time; *t_1/2_*: biological half-life; *C_1min_*: the peak plasma concentration; *V_z_*: volume of distribution; *CL_z_*: clearance. * *p* < 0.05 *vs.* BZP of female rats.

## Abbreviations

The following abbreviations are used in this manuscript:

LC-MS/MS	liquid chromatography coupled to mass spectrometry
BZP	sodium (±)-5-Bromo-2-(α-hydroxypentyl) benzoate
Br-NBP	3-butyl-6-bromo-1(3 *H*)-isobenzofuranone
PHPB	potassium 2-(1-hydroxypentyl)-benzoate
NBP	l-3-n-butylphthalide
PKA	protein kinase A
AKt	protein kinase B
MRM	multiple reaction monitoring
IS	internal standard
LLOQ	lower limit of quantification
QCs	quality control samples
SD	Sprague-Dawley
ULOQ	upper limit of quantification
*AUC*	the area under the plasma concentration–time curve
*MRT*	mean residence time
*t_1/2_*	biological half-life
*C_1 min_*	the peak plasma concentration
*V_z_*	volume of distribution
*CL_z_*	clearance
SFDA	State Food and Drug Administration
SRM	selected reaction monitoring
SIM	selected ion monitoring
ESI	electrospray ionization
ESI-MS	electrospray ionization mass spectrometry
FDA	USA Food and Drug Administration
RSD	relative standard deviation
